# Comparative genomic analyses of *Cutibacterium granulosum* provide insights into genomic diversity

**DOI:** 10.3389/fmicb.2024.1343227

**Published:** 2024-01-17

**Authors:** Peishan Chen, Shaojing Wang, Hongyan Li, Xiaoye Qi, Yuanyuan Hou, Ting Ma

**Affiliations:** ^1^Institute of Integrative Medicine for Acute Abdominal Diseases, Tianjin Nankai Hospital, Tianjin, China; ^2^College of Life Sciences, Nankai University, Tianjin, China; ^3^College of Bioengineering, Tianjin University of Science and Technology, Tianjin, China; ^4^Tianjin JOYSTAR Technology Co., Ltd, Tianjin, China; ^5^College of Pharmacy, Nankai University, Tianjin, China

**Keywords:** *C. granulosum*, genomics, phylogenomic, antibiotic resistance, horizontal gene transfer (HGT)

## Abstract

*Cutibacterium granulosum*, a commensal bacterium found on human skin, formerly known as *Propionibacterium granulosum*, rarely causes infections and is generally considered non-pathogenic. Recent research has revealed the transferability of the multidrug-resistant plasmid pTZC1 between *C. granulosum* and *Cutibacterium acnes*, the latter being an opportunistic pathogen in surgical site infections. However, there is a noticeable lack of research on the genome of *C. granulosum*, and the genetic landscape of this species remains largely uncharted. We investigated the genomic features and evolutionary structure of *C. granulosum* by analyzing a total of 30 Metagenome-Assembled Genomes (MAGs) and isolate genomes retrieved from public databases, as well as those generated in this study. A pan-genome of 6,077 genes was identified for *C. granulosum*. Remarkably, the ‘cloud genes’ constituted 62.38% of the pan-genome. Genes associated with mobilome: prophages, transposons [X], defense mechanisms [V] and replication, recombination and repair [L] were enriched in the cloud genome. Phylogenomic analysis revealed two distinct mono-clades, highlighting the genomic diversity of *C. granulosum*. The genomic diversity was further confirmed by the distribution of Average Nucleotide Identity (ANI) values. The functional profiles analysis of *C. granulosum* unveiled a wide range of potential Antibiotic Resistance Genes (ARGs) and virulence factors, suggesting its potential tolerance to various environmental challenges. Subtype I-E of the CRISPR-Cas system was the most abundant in these genomes, a feature also detected in *C. acnes* genomes. Given the widespread distribution of *C. granulosum* strains within skin microbiome, our findings make a substantial contribution to our broader understanding of the genetic diversity, which may open new avenues for investigating the mechanisms and treatment of conditions such as acne vulgaris.

## 1 Introduction

Acne is a persistent inflammatory skin condition, and its development is intricately linked to factors such as heightened sebum production, excessive skin keratinization, and bacterial overgrowth (Schommer and Gallo, 2013; Zaenglein et al., 2016). To treat acne, a range of antibiotics, including clindamycin, nadifloxacin, ozenoxacin, doxycycline, minocycline, and roxithromycin, are commonly employed (Hayashi et al., 2018). Nevertheless, the presence of antibiotic-resistant bacteria on skin poses a substantial threat to the efficacy of antibiotic treatments. Notably, bacteria carrying the multidrug-resistant plasmid pTZC1, which contains *erm* and *tet* genes responsible for resistance to macrolide-clindamycin and tetracycline, frequently exhibit antibiotic resistance (Nakase et al., 2012, 2020; Aoki et al., 2019, 2020; Koizumi et al., 2022). Consequently, the effective use of antibiotics is intimately connected to the emergence of antibiotic resistance in various skin commensal bacteria, including *Cutibacterium granulosum* (Koizumi et al., 2023b).

*C. granulosum*, formerly known as *Propionibacterium granulosum*, is a gram-positive bacterial species. It belongs to the *Cutibacterium* genus, which encompasses various bacterial species commonly found on human skin. As a part of skin microbiota, *C. granulosum* is primarily located in sebum-rich areas, although it is present at a much lower abundance than *C. acnes* (Park et al., 2020). The coexistence of *C. granulosum* and *C. acnes* has been observed, particularly in acne pustules (Koizumi et al., 2023b). *C. granulosum* exhibits high resistance rates to various antimicrobial agents, potentially posing challenges in treatment if it becomes the causative pathogen of opportunistic infections (Koizumi et al., 2023a). Recent comparative genomic studies have highlighted the transferability of the multidrug-resistant plasmid pTZC1 between *C. granulosum* and *C. acnes*, leading to the accumulation of Antibiotic Resistance Genes (ARGs) and an increased prevalence of multidrug-resistant strains on the skin surface (Koizumi et al., 2023b).

The utilization of Metagenome-Assembled Genomes (MAGs) in in pan-genomic analysis has become increasingly popular in recent years. Tang Li and Yanbin Yin conducted an evaluation of the use of MAGs in pan-genomics analysis by comparing the results of pan-genomic analysis between complete bacterial genomes and simulated MAGs (Li and Yin, 2022). Their findings addressed the impact of incompleteness and contamination on pan-genomic analysis (i.e., incompleteness led to core gene loss, while the contamination had influence on accessory genomes). They recommend utilizing higher quality MAGs, lowering core gene threshold, and employing metagenome mode for gene prediction. Most recently, Marcele Laux and colleagues updated the pan-genome of *Raphidiopsis* by adding newly generated MAGs (Laux et al., 2023). As mentioned in the article, they carefully validated these MAGs by both Average Nucleotide Identity (ANI) and Average Amino acid Identity (AAI) prior to pan-genomic analysis. These studies suggest that high-quality MAGs can be utilized in pan-genomic analysis.

Previous studies have underscored the importance and urgency of conducting more in-depth research on *C. granulosum*. However, there remains a noticeable scarcity of investigations into the genome of *C. granulosum*, leaving its genetic landscape largely unexplored. Thus, the primary objectives of this study are to unravel the genomic diversity and pinpoint the specific biological functions of *C. granulosum* genome, which might improve the understanding of its adaptation in surroundings. To achieve this goal, we conducted comparative genomic analyses, utilizing a total of 30 *C. granulosum* genomes retrieved from both isolates and metagenomes (Supplementary Figure S1).

## 2 Materials and methods

### 2.1 Participant recruitment and sample collection

Participants with good skin condition were recruited at the Tianjin Nankai Hospital, Tianjin, China, between July 15th, 2022 and September 12th, 2022. Ethical approval for the study was received from Tianjin Nankai Hospital research ethics committee in July 2022 (NKYY_YXKT_IRB _2022_040_01). Written informed consents were obtained from all participants. A total of 18 subjects meeting the inclusion criteria were recruited.

The subjects were asked not to clean their skin or apply any lotions, perfumes, cosmetics and other substances to their skin for 24-h prior to the metagenomic sampling. Sterile nonfat cotton swabs were soaked in sterile normal saline, and 4 cm^2^ facial areas of left and right cheeks were wiped for 30 s each. After sampling, the cotton swab heads were placed into sterilized tubes, frozen in liquid nitrogen, and stored at −80°C. The sampling procedure was repeated three times.

### 2.2 DNA extraction and metagenomic sequencing

Genomic DNA was extracted from all the samples within one-week using a Wizard Genomic DNA purification kit (Promega, Madison, WI, United States) according to the manufacturer’s recommended protocol. The quantity and purity of the extracted DNA were determined using a NanoDrop™ 2000 spectrophotometer (Thermo-Fisher Scientific, Waltham, MA, United States). Sequencing libraries were prepared according to the Nextera XT DNA Library Preparation Kit (Illumina) protocol and pooled to 2 mM. Paired-end sequencing run was performed on NextSeq 500 Illumina platform using NextSeq 500 v2 kit with 150 cycles.

### 2.3 Metagenomic data analysis

Adapter removal and quality trimming of the generated raw data were performed using TrimGalore v0.6.0^1^ with default parameters. Host contaminated reads were removed using HoCoRT (Rumbavicius et al., 2023). MAGs were recovered using the metaWRAP pipeline (Uritskiy et al., 2018). Briefly, the modules of “assembly,” “Binning” and “Bin_refinement” were executed step by step with default parameters. The taxonomy lineages for these MAGs were assigned with the classify workflow in GTDB-Tk v.2.1.1 (Chaumeil et al., 2022) by the Genome Taxonomy Database (GTDB) release R207_v2 (Parks et al., 2022). The lineage_wf workflow of CheckM v1.0.18 (Parks et al., 2015) was employed to evaluate the quality of metagenomic bins. Only those *C. granulosum* MAGs with completeness ≥90% and contamination <5% were considered “high quality” (Singleton et al., 2021; Jiang et al., 2022) and retained for downstream analyses.

The MAGs sequences of *C. granulosum* originally generated in this study have been deposited in GenBank under accession numbers JAWMSC000000000 to JAWMST000000000. The corresponding raw reads have also been deposited in GenBank under accession numbers SRR27396937 to SRR27396954.

### 2.4 Public resourced genomes for pan-genome analysis

For comparative analysis, we collected all available genomic sequences for *C. granulosum*, comprising both MAGs and isolate genomes, from the National Center for Biotechnology Information (NCBI) website^2^ as of September 10, 2023. Following this, all the genomes, including those originally presented in this study and those obtained from public database, underwent evaluation using the “lineage_wf” workflow within CheckM v1.07 (Parks et al., 2015). To standardize the genome assembly quality and minimize potential discrepancies in pan-genome analysis, only those genomes meeting the criteria of near completeness (completeness ≥ 90%) and low contamination (<5%) (Singleton et al., 2021; Jiang et al., 2022) were retained for further analysis.

Consequently, a total of 30 genomes of *C. granulosum* were included in this study, comprising 18 MAGs originally generated in this study and 12 retrieved from the NCBI genome database.

### 2.5 Comparative genomic analyses

For all retained genomes of *C. granulosum*, gene predictions were performed using Prokka v1.13 (Seemann, 2014). The resulting GFF3 files served as input for Roary v3.11.2 (Page et al., 2015) to delineate the pan-genome, which comprises the hard-core, soft-core, shell, cloud, and unique genomes. The hard-core genome is defined as the set of genes shared by all 30 *C. granulosum* genomes. The soft-core genome consists of the genes retained by 29 out of the 30 genomes. The shell and cloud genomes encompass gene sets shared by 5–28 and 1–4 genomes, respectively. The unique genome contains the set of genes observed in only one of the genomes.

The phylogenetic inference of *C. granulosum* was reconstructed using the alignment of hard-core genome generated by Roary. To eliminate potential recombination regions, Gubbins v3.0.0 (Croucher et al., 2015) was employed with the following options: “-m 4 -b 4,000 --first-tree-builder fasttree”. Subsequently, a Maximum Likelihood (ML) tree was constructed using RAxML-NG v.1.1 (Kozlov et al., 2019) with rapid bootstrap 1,000 and the best fitting model of “GTR + G4” determined by ModelTest-NG v0.1.7 (Darriba et al., 2020). The final tree was visualized using iTOL (Letunic and Bork, 2021).

### 2.6 Comparative phylogenetic analyses

The 16S rRNA sequences of the 30 *C. granulosum* genomes were identified using Barrnap (0.9-dev, https://github.com/tseemann/barrnap). Multiple Sequence Alignment (MSA) and 16S rRNA-based phylogenetic tree based on ML algorithm were generated by MEGAX software (Kumar et al., 2018) with bootstrap 1,000.

Whole Genome Alignment (WGA) was constructed using the “nucmer” command of MUMmer (Marçais et al., 2018), with *C. granulosum* NCTC11865 (GCA_900186975.1) as the reference genome. MSA was constructed using whole-genome-wide Single Nucleotide Polymorphisms (SNPs) generated by the “show-snps” command, based on the coordinates of the reference genome. Potential recombination regions were eliminated using Gubbins v3.0.0 (Croucher et al., 2015). Subsequently, a WGA-based ML tree was constructed with RAxML-NG v.1.1 (Kozlov et al., 2019), employing rapid bootstrap (1,000 replicates) and the best-fitting model “GTR + G4” determined by ModelTest-NG v0.1.7 (Darriba et al., 2020).

Whole genome SNPs were identified using kSNP4 (Hall and Nisbet, 2023) with the parameters “-k 17 -annotate annotatedGenomes -ML -vcf.” The kSNP4 tool employs an alignment-free approach for SNP identification. The SNPs-based ML tree was generated using FastTree (Price et al., 2010), which was automatically applied in the kSNP4 pipeline.

All phylogenetic trees were visualized using iTOL (Letunic and Bork, 2021).

### 2.7 ANI and AAI

ANI values between all genomes were calculated using fastANI v1.3 (Jain et al., 2018) with the parameter “--fragLen 100.” The size of orthologous regions for ANI calculation were determined by the summation of orthologous matches multiplied by fragLen (100 bp). The pairwise ANI values were plotted using the ggplots package in R.^3^ The AAI values and the proportion of matched CDS were calculated by EzAAI tool with default parameters (Kim et al., 2021).

### 2.8 Functional analyses

The distribution of Clusters of Orthologous Groups (COG) was automated using COGclassifier.^4^

The identification of Clustered Regularly Interspaced Short Palindromic Repeat (CRISPR) regions and CRISPR-associated (Cas) proteins was conducted with CRISPRCasFinder (Couvin et al., 2018), using the genome sequences of *C. granulosum* as input.

The detection of ARGs was performed using the Comprehensive Antibiotic Resistance Database (CARD) due to its consistent maintenance and regular updates (Alcock et al., 2023). Protein sequences of the coding genes predicted in 30 *C. granulosum* genomes were annotated through a BLASTP search against CARD 2023 (Alcock et al., 2023). The search parameters included E-value threshold of less than 1e-5 and minimum alignment length percentage of greater than 40%, as previously described by Zhang et al. (2019).

Putative virulence-related factors within the genomes of *C. granulosum* were identified by searching against the Virulence Factor Database (VFDB) (Liu et al., 2022). Each proteome was individually aligned with the VFDB full dataset using the BLASTp algorithm. A matrix was created based on VFDB hits against proteins in each genome. The matrix was filtered using BLASTp score threshold of ≥80 as described by Rasheed et al. (2017).

### 2.9 Horizontal gene transfer (HGT) analyses

MetaCHIP (Song et al., 2019) was employed to detect HGT among the 30 *C. granulosum* genomes with default parameters. A customized grouping file was provided to MetaCHIP, containing clade-specific information for each genome marked as “clade A,” “clade B,” or “others.” The term “others” represents for the species not located in clade A nor in clade B.

## 3 Results

### 3.1 Genomic overview of *C. granulosum*

This study encompassed a total of 30 *C. granulosum* genomes, consisting of five genomes from pure cultured isolates and the remaining 25 genomes retrieved from metagenomes (Supplementary Table S1). The average genome size of *C. granulosum* is approximately 2.153 ± 0.103 Mb, with an average G + C content of 64.143% ± 0.289% and an average of 1860 ± 94 coding genes per genome. Notably, the genome with the ID GCA_032510525.1, retrieved from metagenomes of infant feces, exhibited a relatively low G + C content of 62.721% compared to the others. All genomes retained in this study demonstrate high assembly quality, with an average completeness of 97.298% ± 2.393% and an average contamination rate of 1.809% ± 1.422%. The average number of contigs for all 25 MAGs is 191, with an average N50 of 72.18 kb.

The distribution of ANI and AAI of MAGs against complete genomes were investigated using both *C. granulosum* TP-CG7 and *C. granulosum* NCTC11865 as references. The average ANI value of 25 MAGs against *C. granulosum* NCTC11865 was 97.35%, with orthologous regions accounting for an average of 88.73% of the *C. granulosum* NCTC11865 genome. For the 25 MAGs against *C. granulosum* TP-CG7, the average ANI value was 98.03%, and the orthologous regions accounted for an average of 89.27% of the *C. granulosum* TP-CG7 genome (Supplementary Table S2). The average AAI value of 25 MAGs against *C. granulosum* NCTC11865 was 97.60%, with matched CDS accounting for an average of 84.49% of the *C. granulosum* NCTC11865 proteome. For the 25 MAGs against *C. granulosum* TP-CG7, the average AAI value was 98.12%, and the matched CDS accounted for an average of 83.96% of the *C. granulosum* TP-CG7 proteome (Supplementary Table S3). Both ANI and AAI values exceed 95%, with a significant portion of the genome/proteome utilized.

### 3.2 Pan-genome determination and distribution of functional categories

**Figure 1:**
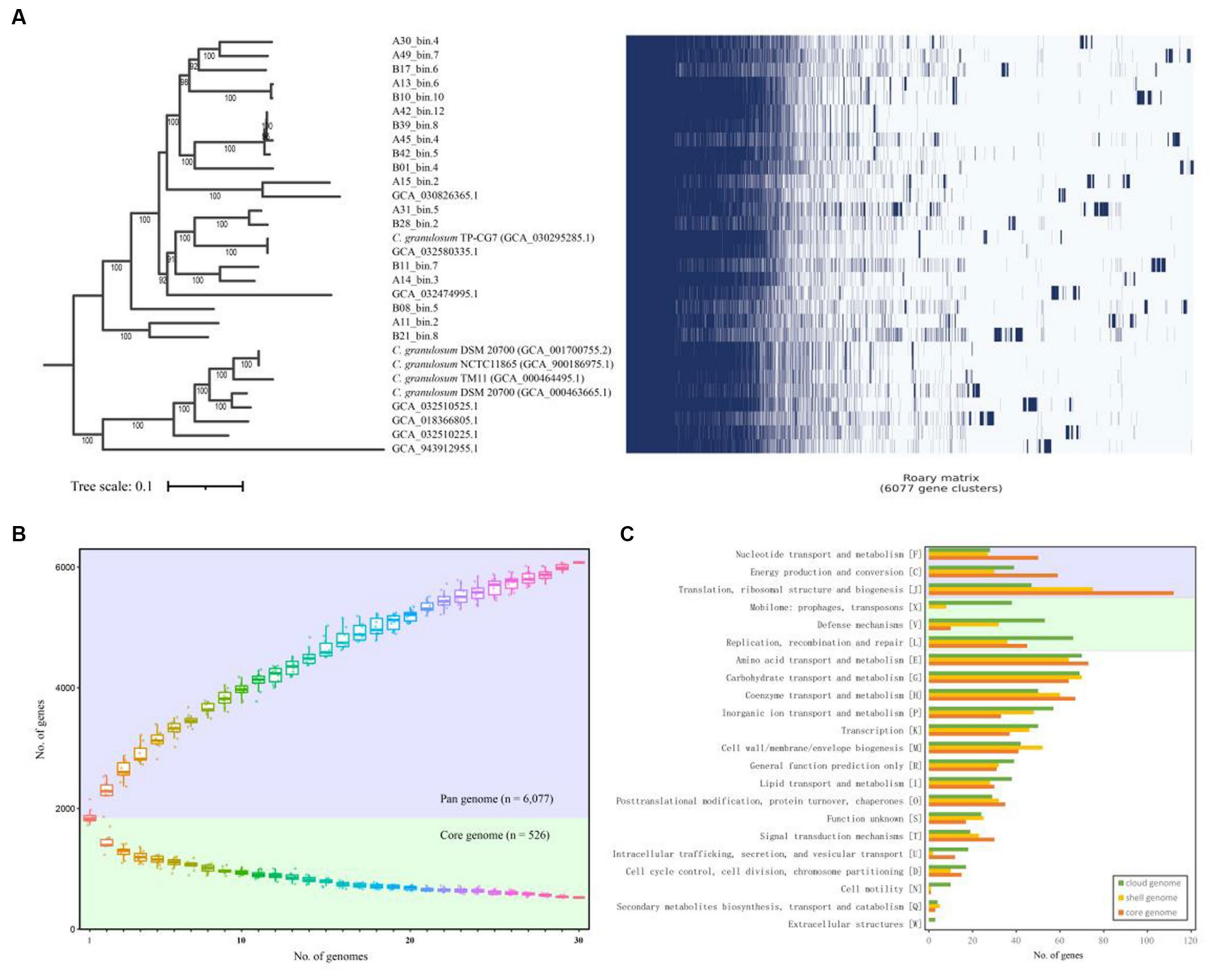
Pan- and core-genome of *C. granulosum*. **(A)** Gene cluster matrix of presence/absence (blue)/(white) of the 6,077 genes (columns) that constitute the pan genome of *C. granulosum* (rows). **(B)** The number of genes increases in the pan genome (blue background) and decreases in the core genome (green background) with the addition of more genomes. **(C)** Number of genes in the core (hard- and soft-core genes), shell and cloud genomes assigned to each functional COG category.

In order to assess genomic conservation across the 30 *C. granulosum* genomes, we conducted a comprehensive pan-genome analysis for defining the sets of hard-core, soft-core, shell, cloud, and unique genomes. A total of 6,077 genes representing the pan-genome of *C. granulosum* were revealed in this study (Figure 1A). Notably, the pan genome did not reach a plateau, as the addition of each new genome continued to increase the number of genes in the pan-genome (Figure 1B).

Among the 6,077 identified coding genes (Supplementary Table S4), 526 genes were shared across all 30 genomes, categorizing them as “hard-core” genes, while 412 genes were shared by at least 29 genomes, classifying them as ‘soft-core’ genes. The analysis also revealed 1,348 “shell” genes, which were shared by 5–28 genomes, and 3,791 “cloud” genes, shared by 1–4 genomes. Notably, the “cloud” genes constituted 62.38% of the pan-genome. It was observed that approximately 40% (2,443 out of 6,077) of the pan-genome consisted of “unique” genes, observed in only one of these genomes. The presence of “shell” and “cloud” genomes, especially the “unique” genome, contributes significantly to the genetic diversity and, presumably, phenotypic differences among strains.

Functional categories were assigned using COGclassifier, a tool designed for classifying prokaryotic protein sequences into COG functional categories. The number of genes for each functional category was represented with bar graphs (Figure 1C). Three basic biological functional categories were enriched in core-genome (union of hard- and soft-core genomes), i.e., nucleotide transport and metabolism [F], energy production and conversion [C] and translation, ribosomal structure and biogenesis [J]. Another three functional categories of mobilome: prophages, transposons [X], defense mechanisms [V] and replication, recombination and repair [L] were enriched in the cloud genome. The “cloud” genome is the set of genes shared by four genomes or fewer, making up a significant portion (62.38%) of the pan-genome for *C. granulosum*.

### 3.3 Phylogenomic analysis

**Figure 2:**
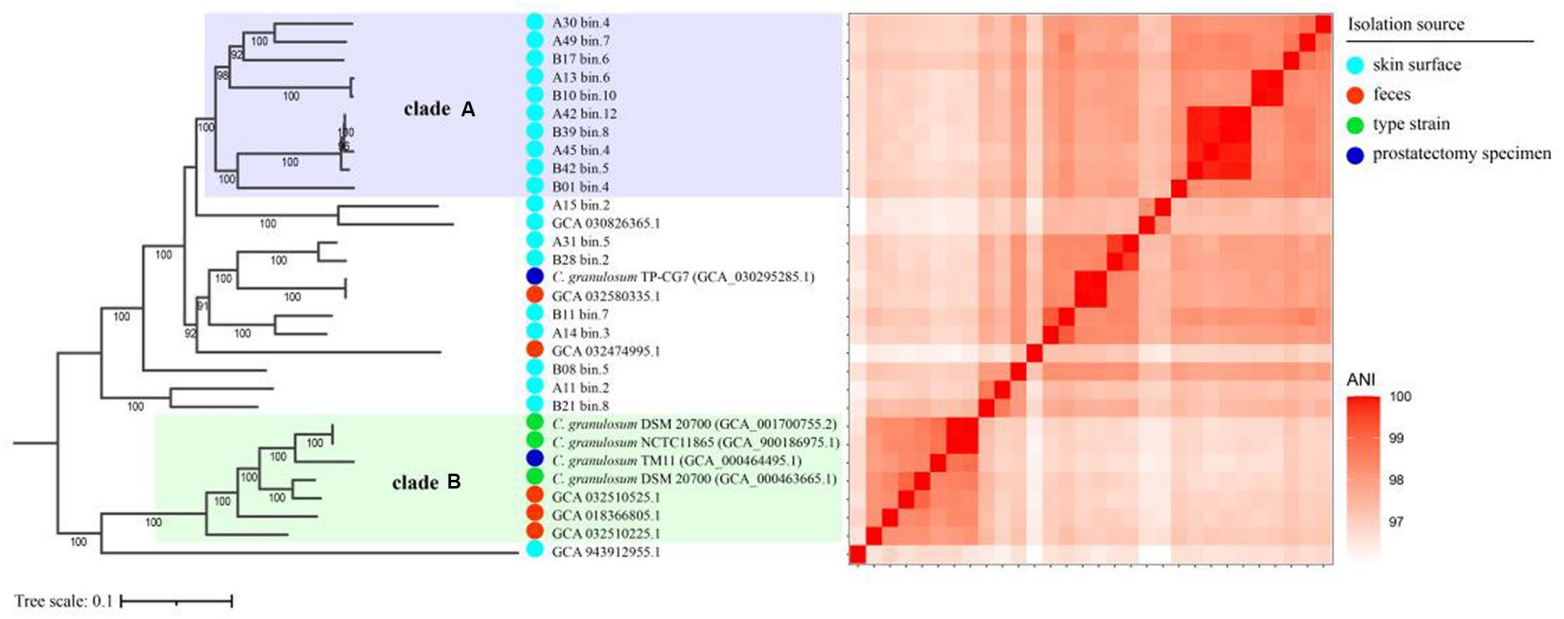
Phylogenomic analysis of *C. granulosum*. The phylogenetic tree reconstructed using core genome. The heatmap pairwise the distribution of pairwise ANI values across the phylogenetic tree.

To investigate the relationships between the *C. granulosum* genomes generated in this study and those available in public database, the core genome was used to reconstruct the phylogenetic tree. The most striking outcome of the phylogenomic analysis was the highly diverse relationships observed within *C. granulosum*. As depicted in Figure 2, the phylogenetic tree revealed two distinct monophyletic clades. Clade A consisted of ten genomes originally retrieved from skin surface of adult volunteers living in Tianjin, China. Clade B was comprised of seven genomes retrieved from isolates (*n* = 4, type materials) and metagenomes (*n* = 3, samples of infant feces collected at Magee-Womens Hospital of UPMC, Pittsburgh, United States).

The 16S rRNA sequences were identified in all 5 isolate genomes and only one MAG (Supplementary Figure S2). Owning to the miss identification of 16S rRNA in mostly MAGs, the identification of clade A and clade B was verified using WGA-based phylogenetic tree (Supplementary Figure S3) and SNPs-based phylogenetic tree (Supplementary Figure S4). Consistent monophyletic clades A and B, representing the same genomes, were observed in the phylogenetic trees based on WGA, SNPs and core genome (Figure 2; Supplementary Figures S3, S4).

**Figure 3:**
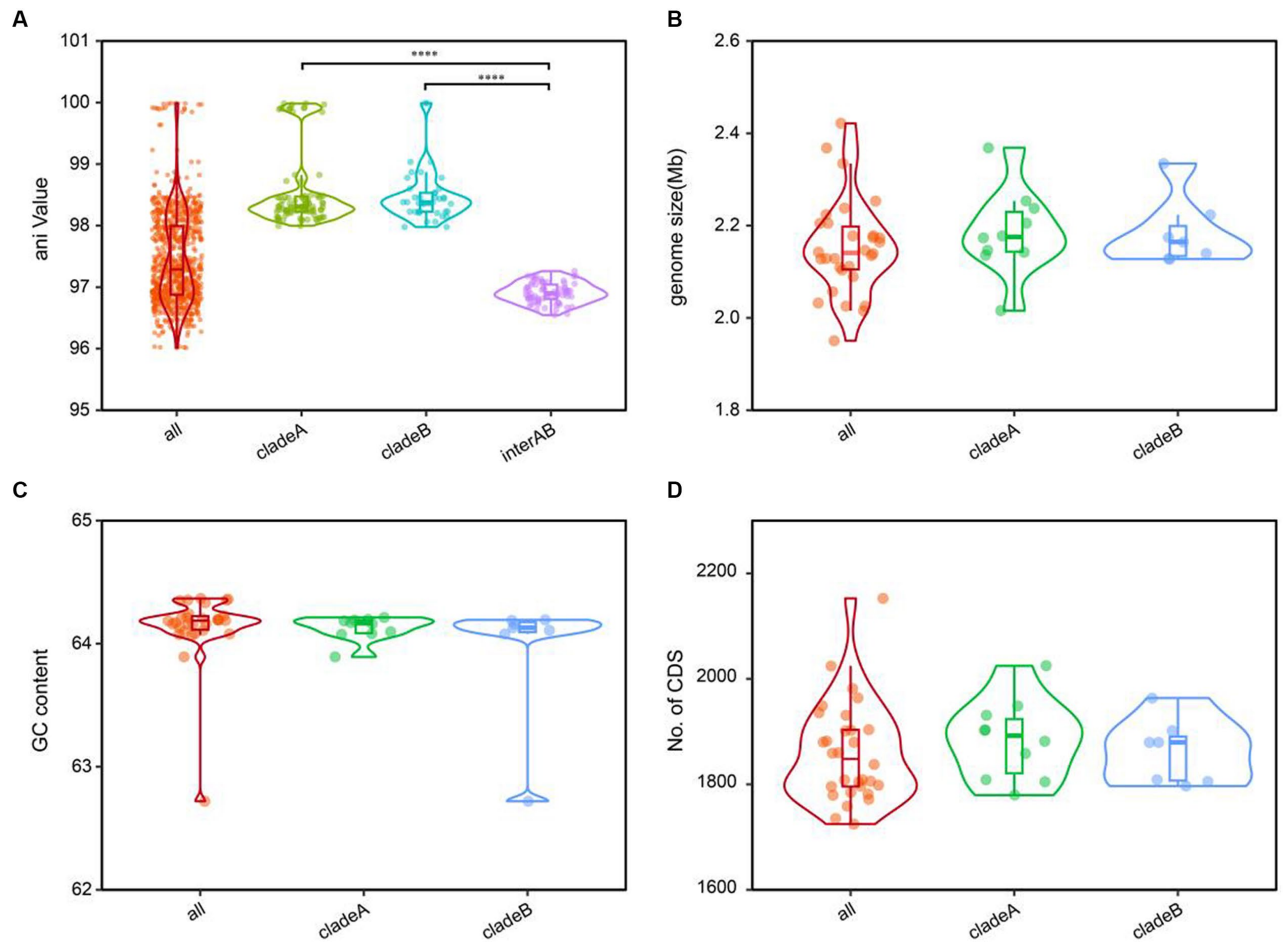
Comparative genomic features of *C. granulosum*. **(A)** Comparation of pairwise ANI values. **(B)** Comparation of genome size. **(C)** Comparation of genomic G+C content. **(D)** Comparation of protein coding genes number.

The cladistic relationships and genomic diversity were further validated using the ANI approach, a robust measure of genomic relatedness between strains (Figures 2, 3A). The overall ANI values across *C. granulosum* averaged at 97.45% ± 0.74%. Notably, the intra-clade ANI values of genomes in Clade A and Clade B were 98.55% ± 0.61 and 98.47% ± 0.42%, respectively. However, the inter-clade ANI values between genomes in Clade A and B were notably lower, measuring only 96.91% ± 0.16%. These significant differences in ANI values between intra-clade and inter-clade comparisons (Figure 3A, *p* < 0.0001) highlight the genetic diversity among these strains. Although no differences were observed in other genomic features such as genome size, G + C content, and the number of protein-coding genes (Figures 3B–D), the distant relationships between *C. granulosum* isolates and the larger proportion of cloud genome within the pan-genome offer a valuable perspective on the acquisition of biological functions.

A total of 64 interclade HGT events were observed (Supplementary Table S5), including 48 HGT events between species in clade A and others, and 16 HGT events between species in clade B and others. No HGT event was observed between species located in “clade A” and “clade B.” Besides, a total of 1,463 clade-specific genes were observed, comprising 799 genes identified only in strains within Clade A and 664 genes identified only in strains within Clade B. The comparation of COG categories distribution for clade-specific genes was showed as Supplementary Figure S5. The results revealed that specific genes in Clade A were enriched in the categories of “[L] Replication, recombination and repair,” “[X] Mobilome: prophages, transposons,” “[C] Energy production and conversion” and “[O] Posttranslational modification, protein turnover, chaperones,” compared to those in Clade B (two-fold change). Meanwhile, specific genes in Clade B were enriched in the categories of “[I] Lipid transport and metabolism,” “[G] Carbohydrate transport and metabolism,” “[J] Translation, ribosomal structure and biogenesis,” “[V] Defense mechanisms.” Additionally, a total of 1,284 clade A-specific SNPs and 4,292 clade B-specific SNPs were identified (Supplementary Table S6).

### 3.4 Functional analysis of *C. Granulosum* genomes

To gain insights into the functional profiles of *C. granulosum*, the distributions of putative ARGs, virulence-related factors, and CRISPR-Cas systems were investigated.

**Figure 4:**
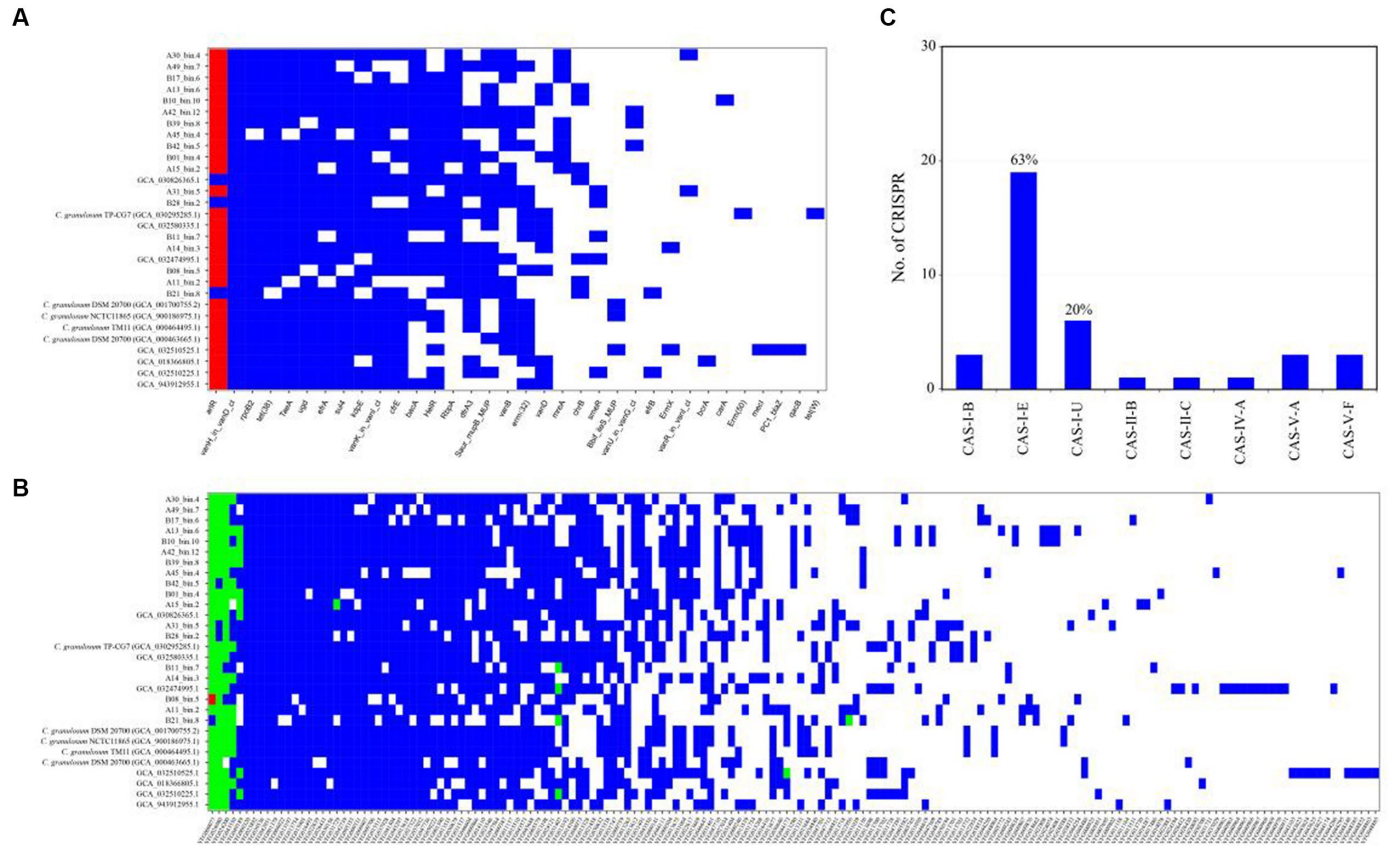
Distribution of functional genes in *C. granulosum* genomes. **(A)** Distribution of ARGs based on CARD. Blue markings denote the existence of ARGs within the corresponding genome, whereas red dots indicate the presence of two copies of the respective ARGs within the genome. **(B)** Distribution of virulence factors based on VFDB. Blue markings denote the existence of presumed virulence factors, whereas green and red dots indicate the presence of two or three copies of presumed virulence factors within the genome, respectively. **(C)** Occurrence and diversity of CRISPR-Cas systems.

The ARGs were identified through a BLASTp search against the CARD database. The CARD database is a meticulously curated resource comprising antibiotics, their targets, ARGs, associated proteins, and antibiotic resistance literature. The analysis revealed a wide range of potential ARGs within the *C. granulosum* genomes, suggesting its potential tolerance to various environmental challenges (Figure 4A; Supplementary Table S7).

Putative virulence-related factors were identified based on the VFDB database, known for its comprehensive coverage of virulence factors and detailed information, including structural features, functions, and mechanisms. Our analysis revealed approximately 72 ± 8 genes encoding for virulence-related factors in each *C. granulosum* genome (Figure 4B; Supplementary Table S8).

CRISPR-Cas systems constitute the bacterial adaptive immune system, providing resistance against bacteriophage infections (Barrangou et al., 2007). This system is known for its adaptability and heritability. In our study, we investigated the presence and diversity of CRISPR-Cas systems across the 30 *C. granulosum* genomes. Subtype I-E of the CRISPR-Cas system was identified in 63% (19 out of 30) of the genomes (Figure 4C), based on the presence of the corresponding signature *cas* genes.

## 4 Discussion

*Cutibacterium* strains, particularly *C. granulosum* and *C. acnes*, are crucial members of human skin microbiome and play vital roles in both skin health and disease (Cobian et al., 2021; Koizumi et al., 2023b). Recently, the study by Juri Koizumi and colleagues emphasized the significance of *C. granulosum* in skin health and disease by demonstrating the horizontal transfer of the plasmid pTZC1 between *C. acnes* and *C. granulosum* strains (Koizumi et al., 2023b). This multidrug resistance plasmid pTZC1, which carries genes for macrolide-clindamycin resistance (*erm*(50)) and tetracycline resistance (*tet*(W)), can lead to antimicrobial resistance.

*C. acnes* is the most extensively studied species, and a substantial number of corresponding genomes have been sequenced and are accessible in public databases. Currently, the NCBI genome database^5^ contains a total of 497 genomes of *C. acnes*. A previous study by Natalia Cobian and colleagues involved a comparative genomic analysis of 255 *C. acnes* genomes (Cobian et al., 2021). These results revealed the genomic landscape of *C. acnes* including pan-genome, distinct phylogenetic clades and diverse CRISPR-Cas systems.

In this study, we present a comparative genomic analysis of *C. granulosum*, utilizing MAGs and genomes of isolated strains. The pan-genome depiction of gene presence and absence unveiled accessory genes and gene groups contributing to *C. granulosum*’s functional diversity. Our findings revealed a relatively small and open pan-genome comprising 6,077 genes, with a substantial “cloud” genome accounting for 62.38% of the pan-genome. Remarkably, for the first time, we observed two distinct phylogenetic clades representing strains retrieved from distant environments, a pattern somewhat similar to the genomic diversity found in *C. acnes* (Cobian et al., 2021). Our research significantly augments the dataset of *C. granulosum* genomes and provides an encompassing genomic landscape through pan-genome analysis and functional assessment.

Based on the gene distribution across COG categories, we could infer the genetic diversity of *C. granulosum* in terms of transposons, recombination, and defense mechanisms. While it remains challenging to directly associate these clades with specific ecological niches due to the limited number of genomes available at present, the results presented here highlight the genomic diversity of *C. granulosum*. The data provided in this study significantly contributes to the understanding of this species and expands the research foundation for future studies. The prevalence of CRISPR-Cas systems subtypes I-E in *C. granulosum*, as also detected in *C. acnes* genomes (Cobian et al., 2021), offers insights into likely shared strain divergence and adaptive differentiation between these two species. Furthermore, our results serve as a foundational reference and present new prospects for modulating the composition of skin microbiota using naturally occurring phages, engineered phages, and/or heterologous CRISPR-Cas systems.

The knowledge presented here is essential for understanding the role of *C. granulosum* in skin ecosystem and its potential applications, including the possibilities of utilizing *C. granulosum* to maintain skin health, advance biotechnological applications, and foster innovation in the fields of cosmetics and pharmaceuticals. Future research endeavors will continue to unveil the precise role of *C. granulosum* in promoting skin health and maintaining microbial balance, thus accelerating developments in its various application areas. While this study has made a significant contribution by substantially expanding the number of *C. granulosum* genomes and including high-quality accessible genomes from public databases, it is worth noting that the overall available genomic data for this species remains relatively limited.

## 5 Conclusion

Given the widespread distribution of diverse *C. granulosum* within skin microbiome, whole-genome sequencing offers valuable insights into its roles in health and disease. Comparative genomics analyses provide a robust method for examining extensive genome datasets. Our findings significantly contribute to the broader understanding of the genetic diversity within *C. granulosum*. Notably, our study is the first to reveal the presence of two distinct phylogenetic clades based on genomic data. Understanding the differential genetic content among *C. granulosum* strains in future research may open new avenues for investigating the mechanisms and treatment of conditions such as acne vulgaris.

## Data availability statement

The datasets presented in this study can be found in online repositories. The names of the repository/repositories and accession number(s) can be found in the article/Supplementary material.

## Author contributions

PC: Data curation, Formal analysis, Methodology, Writing – original draft, Writing – review & editing. SW: Data curation, Formal analysis, Methodology, Writing – original draft, Writing – review & editing. HL: Data curation, Validation, Writing – review & editing. XQ: Formal analysis, Writing – review & editing. YH: Formal analysis, Writing – review & editing. TM: Formal analysis, Methodology, Resources, Validation, Writing – original draft, Writing – review & editing.

## Footnotes

^1^ https://github.com/FelixKrueger/TrimGalore

^2^ www.ncbi.nlm.nih.gov

^3^ www.r-project.org

^4^ https://github.com/moshi4/COGclassifier

^5^ www.ncbi.nlm.nih.gov/genome
