# Supplementary figures and images for: Comparative genomic analyses of Cutibacterium granulosum provide insights into genomic diversity
Source: Front Microbiol. 2024 Jan 17;15:1343227. doi: 10.3389/fmicb.2024.1343227 (PMC10832045; doi:10.3389/fmicb.2024.1343227)

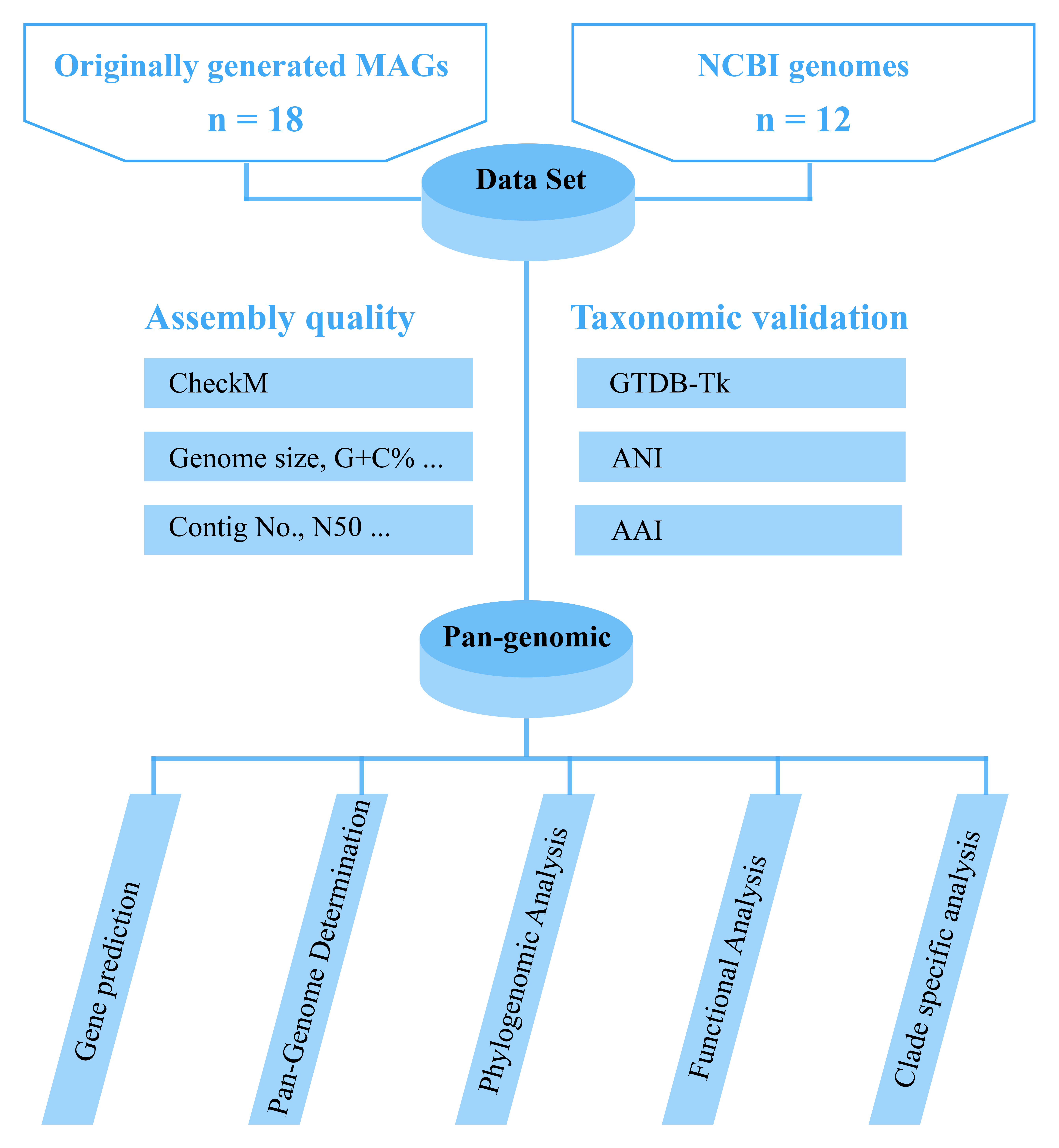

Supplement: Supplementary file 10 [file Image_1.JPEG]

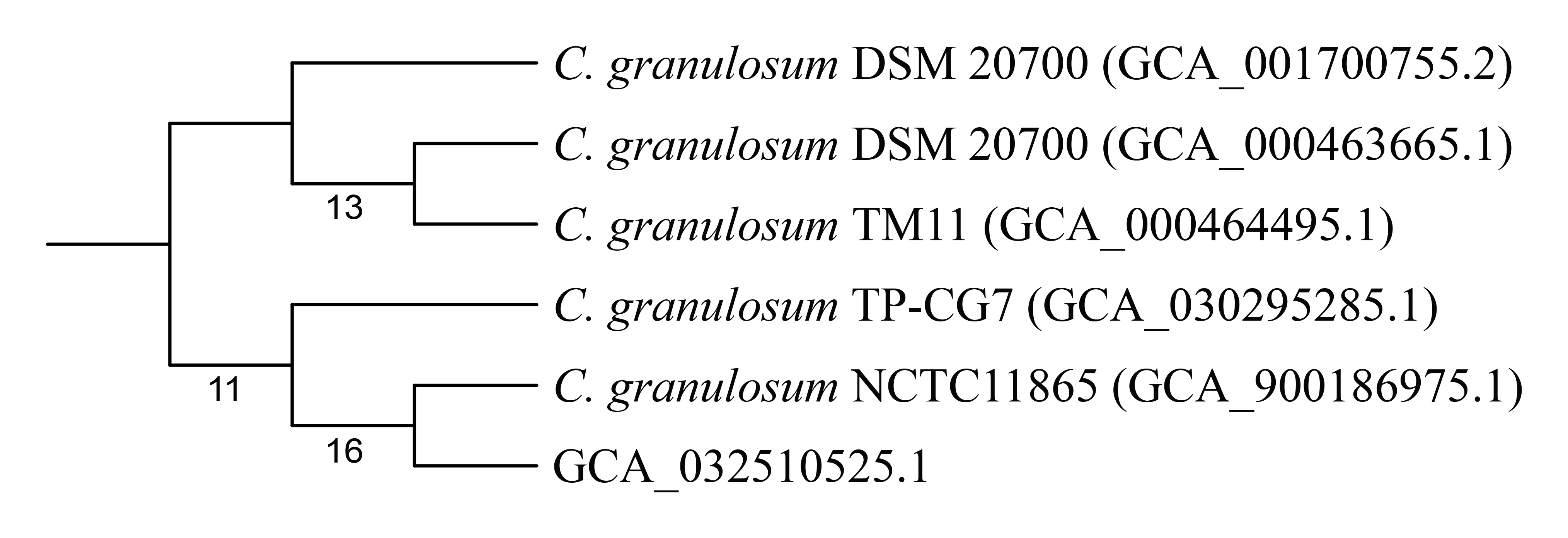

Supplement: Supplementary file 11 [file Image_2.JPEG]

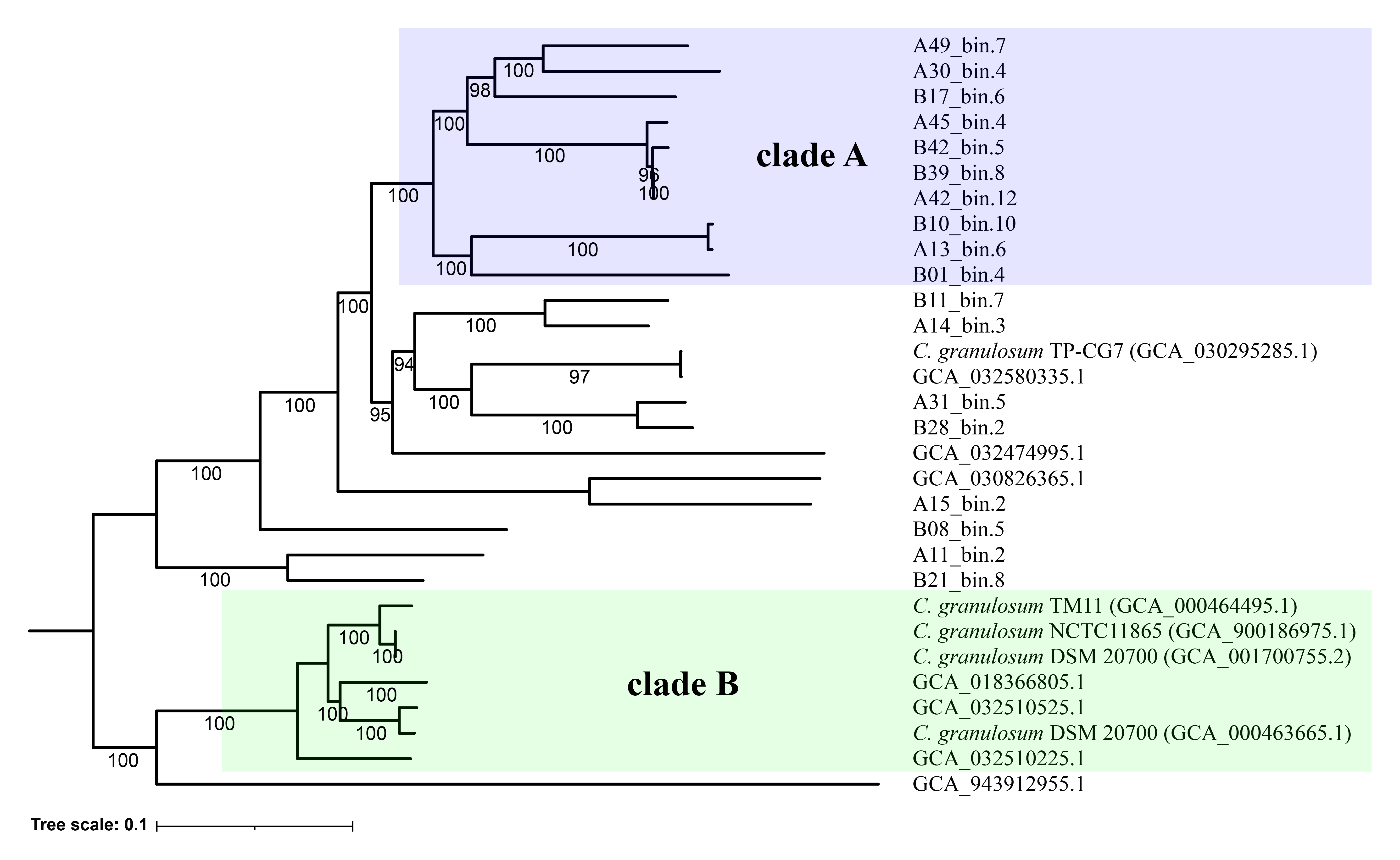

Supplement: Supplementary file 12 [file Image_3.JPEG]

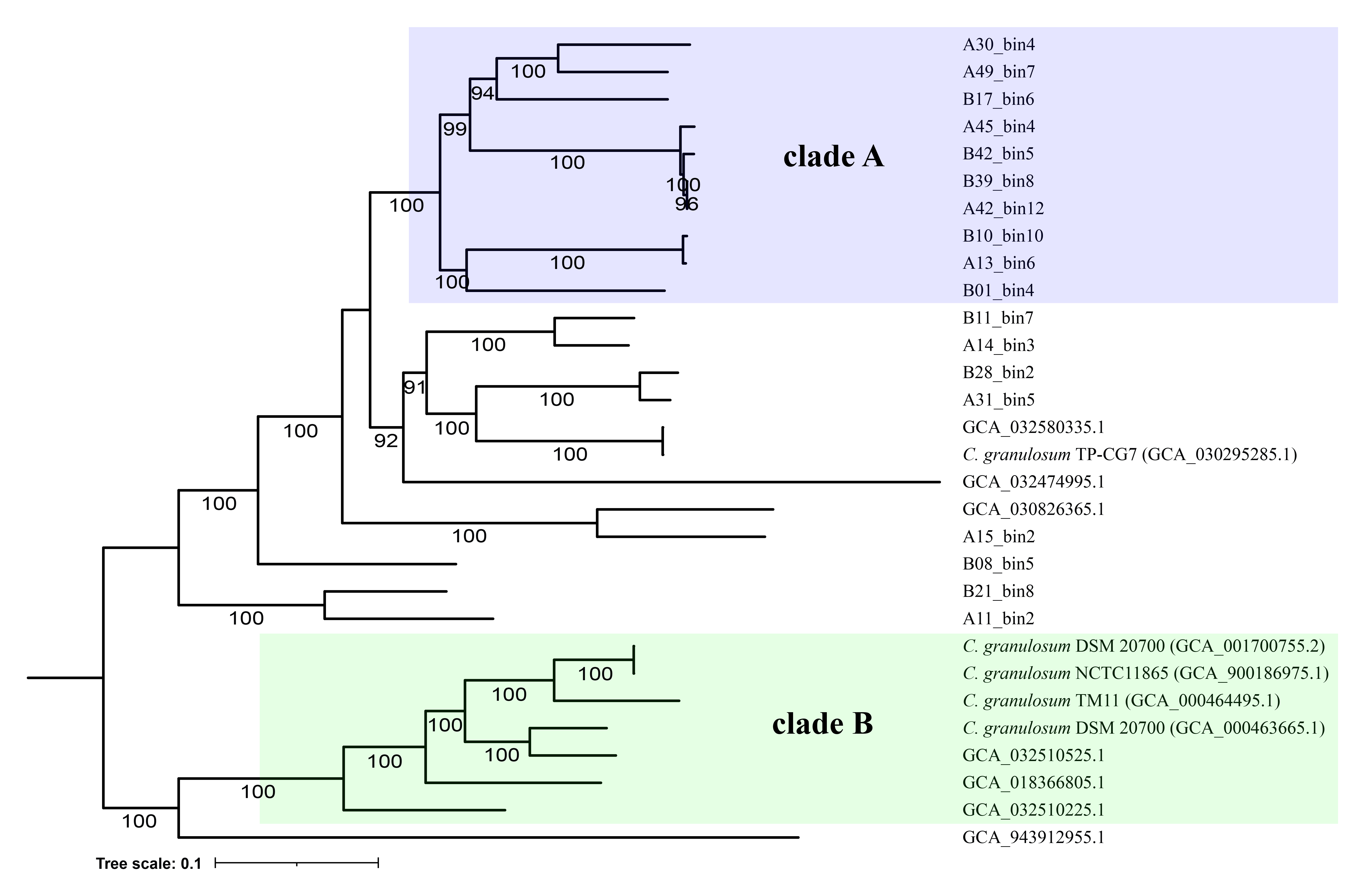

Supplement: Supplementary file 13 [file Image_4.JPEG]

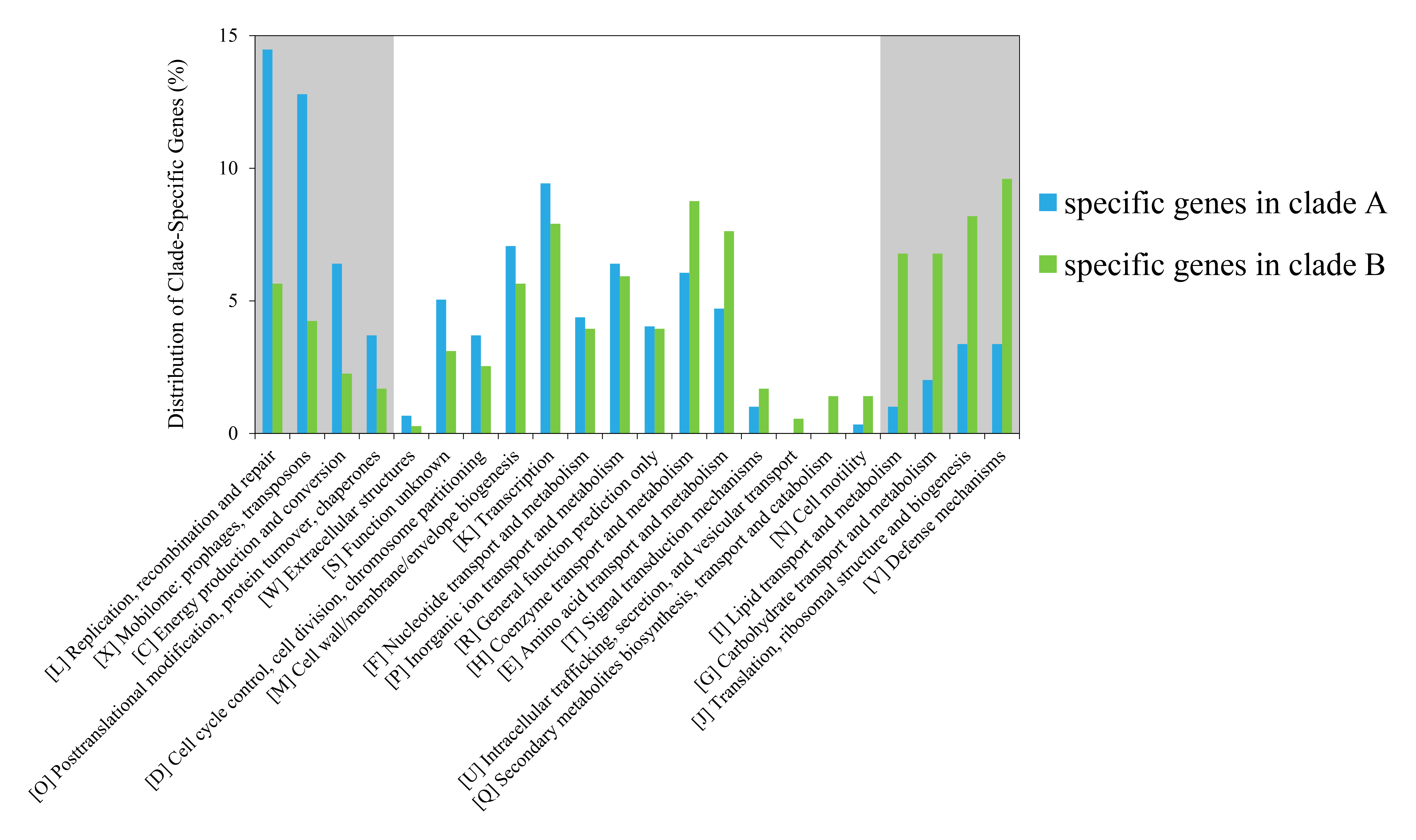

Supplement: Supplementary file 14 [file Image_5.JPEG]
